# Mediators of the Effect of Obesity on Stroke and Heart Disease Risk: Decomposing Direct and Indirect Effects

**DOI:** 10.2188/jea.JE20210476

**Published:** 2023-10-05

**Authors:** Yongho Jee, Mikyung Ryu, In Sun Ryou, Joung Hwan Back, Sung-il Cho, Seung Sik Hwang

**Affiliations:** 1 Advanced Biomedical Research Institute Ewha Womans University Seoul Hospital, Seoul, Republic of Korea; 2 Department of Sports and Health Science, College of Human-Centered Convergence, Kyonggi University, Suwon, Republic of Korea; 3 Department of Family Medicine, Ewha Womans University Seoul Hospital, Seoul, Republic of Korea; 4 Health Insurance Policy Research Institute, Wonju, Republic of Korea; 5 Department of Public Health Science, Graduate School of Public Health, Seoul National University, Seoul, Republic of Korea

**Keywords:** body mass index, mediator, cohort, cardiovascular risk

## Abstract

**Background:**

The prevalence of overweight and obesity are well known risk factors of atherosclerotic cardiovascular disease (ASCVD). We aimed to examine the association between body mass index (BMI) and ASCVD over a 23-year follow-up in young adults. We also qualified how much of the effects of obesity on ASCVD were mediated through blood pressure, cholesterol, and glucose.

**Methods:**

Data are from the Korean Life Course Health Study, a cohort study of 226,955 Korean young adults aged 20–39. At baseline, the participants undertook routine health assessments where their BMI was measured in 1992–1994; and the metabolic mediators including systolic blood pressure (SBP), fasting serum glucose (FSG), and total cholesterol (TC) were re-measured in 2002–2004. The main outcomes of the study include incident events of ischemic heart disease (IHD), stroke, and ASCVD between 2005 and 2015. Cox proportional model was used to calculate adjusted hazard ratios (HRs) for ASCVD.

**Results:**

In both men and women, the direct effect of BMI on ASCVD was greater than the indirect effect. The percentage of excess HR of BMI mediated by all of the metabolic mediators, including SBP, FSG, and TC, was 45.7% for stroke and 18.7% for IHD in men and 27.5% for stroke and 17.6% for IHD in women.

**Conclusion:**

High BMI in young adults increases the risk of metabolic mediators in their middle age, and metabolic mediators explain the adverse effects of high BMI on stroke risk than IHD risk.

## INTRODUCTION

Although Asian populations, in general, have lower body mass index (BMI) than non-Asian populations, the demographic and nutrition transition accompanied by rapid economic growth has inevitably generated marked changes in dietary habits and lifestyles of Asian populations. For example, South Korea’s economy grew at a rapid pace during the past 3 decades, the economy has improved, people’s incomes have increased, and westernized dietary habits have become popular, just like other developing countries that experienced nutrition and health transitions during an industrialization period. Since then, unfavorable shifts in lifestyles leading to weight gain among Korean population have been reported in several cross-sectional studies.^1^^,^^2^

The World Health Organization proposed that the definition of obesity should be different for Asians from that for Europeans. The suggested categories are as follows: 18.5–22.9 kg/m^2^ as normal weight; 23–24.9 kg/m^2^ as overweight; 25–29.9 kg/m^2^ as moderate obesity; and over 30 kg/m^2^ as severe obesity.^3^ High BMI is an important risk factor for intermediate risk factors, such as hypertension, diabetes, and dyslipidemia, which also increase the risk of cardiovascular disease (CVD).^4^^–^^7^ High BMI in young adults is also associated with increased risk of CVD in their middle age.^8^^–^^14^

Several mechanisms for the association of BMI with heart disease have been suggested in western countries that aimed to partially divide the effect of obesity on CVD into direct and indirect effects. However, the mechanisms might be different in Asian populations, where the average BMI is lower than that of other populations. Previous studies on metabolic mediators, including blood pressure, fasting glucose, and total cholesterol, on CVD used baseline measurements of mediators without accounting for the time-varying nature of these variables and did not consider the subtypes of atherosclerotic cardiovascular disease (ASCVD).

In this study, we examined the association between BMI and ASCVD over a 23-year follow-up in young adults. Specifically, we investigated whether the participants with high BMI in their 20s had increased risk of ASCVD, including stroke and ischemic heart disease (IHD), later in their 40s. We also decomposed the effects of BMI on ASCVD into total effect, direct effect, and indirect effect through metabolic mediators in their 30s.

## METHODS

### Study population

The Korean Life Course Health Study (KLCHS) is a cohort from the Korean Medical Insurance Corporation (KMIC) on beneficiaries who were government employees, as well as private school teachers. Among the entire Korean population (approximately 43.7 million in 1992), 4,662,438 (10.7%) were insured by KMIC, 1,297,833 workers and their 3,364,605 dependents. All participants were required to participate in biennial medical examinations, and approximately 94% of these participants completed their examinations either in 1992 or 1994.^15^

The KLCHS cohort consists of 430,951 young Korean adults (307,652 men and 123,299 women) between the ages of 20 and 39 years who received health insurance from the KMIC with biennial medical evaluations in 1992 and 1994 (Table 1). Among these young adults, 205,840 (67.0%) were enrolled in 1992, and 101,201 (33.0%) were enrolled in 1994. The exclusion criteria among 430,951 participants were as follows: those who had missing data on height, systolic blood pressure (SBP), fasting serum glucose (FSG), total cholesterol (TC), or BMI (*n* = 71,760); those who had missing data on smoking status, exercise, or alcohol drinking (*n* = 2,091); those who had a history of cancer or ASCVD before recruitment (*n* = 6,170); and those who died in the period of time between their questionnaire completion and the follow-up initiation on 1^st^ of January in the subsequent year. Therefore, we analyzed 169,429 participants for this study. We received approval for our study proposal from the Institutional Review Board of Human Research, Yonsei University (4-2001-0029), and the Seoul National University (E1812/001-010). This was a retrospective cohort study using past routine laboratory data, and thus consent was waived.

**Table 1.** Baseline and repeated measurements of study participants

Characteristic	Men			Women		
	1992–1994	2002–2004		1992–1994	2002–2004	

	*N* = 98,117 (B)	*N* = 98,117 (A)	A − B	*N* = 71,312 (B)	*N* = 71,312 (A)	A − B
Age, years	26.8 (2.0)	36.3 (2.4)		25.3 (2.6)	34.9 (3.1)	
Body mass index, kg/m^2^	22.4 (2.4)	24.3 (2.9)	1.9	20.3 (2.0)	21.5 (2.5)	1.2
Systolic blood pressure, mm Hg	120.0 (11.6)	123.0 (13.7)	3.0	111.3 (10.3)	111.7 (12.1)	0.4
Fasting serum glucose, mm Hg	86.3 (13.6)	91.6 (20.6)	5.3	83.3 (11.7)	86.1 (12.5)	2.7
Total serum cholesterol, mg/dL	173.9 (33.0)	194.7 (36.0)	20.8	172.9 (33.0)	178.8 (32.3)	5.9
Alcoholic drinks, g per day	17.1 (23.6)	14.3 (21.0)	−2.8	1.05 (3.8)	0.9 (2.9)	0.06
Smoking status, %						
Former smoker	13.2	17.7	4.5	0.5	0.6	0.1
Current smoker	65.1	47.4	−17.7	0.1	0.2	0.1
Any alcohol use (yes), %	83.3	75.6	−7.7	32.3	23.8	−8.5
Physical activity (yes), %	20.4	62.6	42.2	8.7	29.4	20.7

^*^Data are expressed as mean (standard deviation) unless otherwise indicated. All differences (A − B) were statistically significant (*P* for paired t test: <0.001).

### Data collection

The KMIC biennial examinations were conducted by medical staff at local hospitals based on a standardized procedure between 1992 and 2004, and participants were asked to describe their lifestyles, including smoking habits and alcohol consumption. In the study, participants were classified as current smokers if they reported to be a smoker at baseline for at least 1 year, never smokers if they had never smoked, and ex-smokers if they used to smoke but not anymore. BMI was calculated as weight/height^2^ (kg/m^2^) and was categorized into six groups (<18.5, 18.5–21.4, 21.5–24.9, 25.0–27.9, 28.0–31.9, or ≥32.0 kg/m^2^).^16^^,^^17^ We defined BMI over 25 kg/m^2^ as overweight and BMI over 30 kg/m^2^ as obesity.

### Morbidity and mortality follow-up

**Figure 1.**
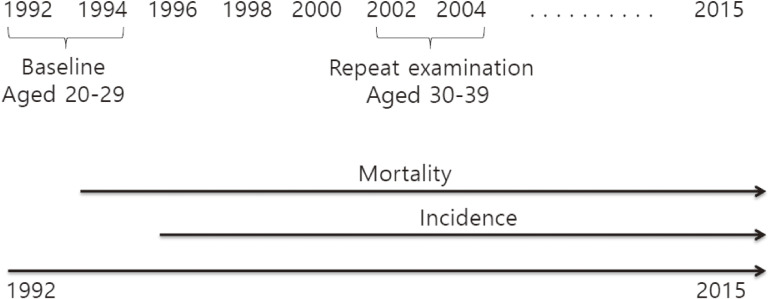
Time Timeline for data collection in the Korean Life Course Health Study, 1992–2015.

Our main outcome variables include morbidity from (1) IHD (ICD-10 codes I20–I25), (2) stroke (codes I60–I69), and (3) total ASCVD, including hypertensive diseases (codes I10–I15), IHD (I20–I25), hemorrhagic stroke (I60–I62), thrombotic stroke (I63), other types of stroke (I64–I69), other heart diseases related to ASCVD (I44–I51), sudden death (R96), and other vascular diseases (I70–I74) between 2005 and 2015. We used the first event in our analyses for those who had more than one events during the follow-up period. In our data, professionally trained and certified medical chart recorders organized charts and assigned discharge diagnoses. In terms of morbidity and mortality, follow-up was 100% complete because all participants were followed by electronic linkage to national databases for the first ASCVD events. The follow-up period was up to 23 years from January 1, 1993 to December 31, 2015 (Figure 1).

### Statistical analysis

We used Cox proportional hazards models to estimate hazard ratios (HRs) and 95% confidence intervals (Cis) of the associations between baseline BMI and ASCVD events in men and women, adjusting for the following covariates: age at enrollment (continuous variable), alcohol intake (yes and no), smoking status (non, ex, current), and participation in regular exercise (yes or no). The Cox proportional model, which examined the effects of young adults’ BMI measured in 1992–1994 on ASCVD, adjusted baseline covariates and additionally adjusted metabolic mediators, including SBP, FSG, and TC examined in 2002–2004. We used the mediation proposed by Vanderweele,^18^^,^^19^ which IS based on causal inference principles, to estimate the total effect, direct on ASCVD by obesity, and indirect effect mediated by metabolic mediators on ASCVD. We employed a counterfactual framework, which was previously defined by Robins and Greenland.^20^ We also calculated the percentage of excess HR mediated based on the direct and indirect effects. All analyses were conducted using SAS software, version 9.3 (SAS Institute, Cary, NC, USA).

## RESULTS

The mean BMI at baseline (1992–1994) was 23.0 kg/m^2^ in men and 20.9 kg/m^2^ in women, which increased to 24.3 kg/m^2^ and 22.0 kg/m^2^, respectively, after 10 years of follow-up (2002–2004). During this period, SBP, FSG, and TC also increased in both men and women. In particular, TC increased by 15.1 mg/dL for men and 11.1 mg/dL for women. Baseline prevalence of current smoking and alcohol use in men decreased over the 10 years (63.0% to 42.4% and 79.7% to 75.6%, respectively), whereas prevalence of physical activity increased by 45.5% in men and 31% in women (Table 1). For both men and women higher body mass index at baseline (1992–1994) showed statistically significant correlation (eTable 1) and association with mediators (2002–2004) (eTable 2).

For the entire follow-up period (1993–2015), there were 4,827 ASCVD events in men and 1,541 events in women. After 10 years of follow-up from baseline (2005–2015), there were 3,469 ASCVD events in men and 934 events in women.

In both sexes, BMI showed a linear relationship with the occurrence of ASCVD. In addition, all three mediators (SBP, FBS, TC) showed a positive association with the occurrence of ASCVD (Table 2).

**Table 2.** Hazard ratios for ASCVD during follow-up (2005–2015), according to BMI after adjusting for baseline covariates and intermediate variables

	Men (*N* = 97,657)			Women (*N* = 71,133)		

	Model 1	Model 2	Model 3	Model 1	Model 2	Model 3
	HR (95% CI)	HR (95% CI)	HR (95% CI)	HR (95% CI)	HR (95% CI)	HR (95% CI)
**Baseline (1992**–**1994)**						
BMI						
<18.5	1.0	1.0	1.0	1.0	1.0	1.0
18.5–21.4	1.25 (0.94–1.66)	1.17 (0.87–1.55)	1.13 (0.85–1.51)	1.18 (0.97–1.43)	1.14 (0.94–1.38)	1.11 (0.92–1.35)
21.5–24.9	1.74 (1.31–2.31)	1.47 (1.10–1.95)	1.34 (1.01–1.79)	1.52 (1.23–1.88)	1.38 (1.11–1.71)	1.27 (1.03–1.58)
25.0–27.9	2.63 (1.97–3.52)	1.94 (1.45–2.60)	1.65 (1.23–2.22)	2.08 (1.39–3.11)	1.80 (1.20–2.70)	1.55 (1.03–2.33)
28.0–31.9	3.87 (2.81–5.33)	2.61 (1.89–3.60)	2.11 (1.53–2.92)	2.12 (0.78–5.72)	1.83 (0.67–4.96)	1.50 (0.55–4.06)
≥32.0	5.66 (3.33–9.60)	3.42 (2.00–5.82)	2.55 (1.49–4.36)	4.15 (0.58–29.60)	3.46 (0.48–24.68)	2.53 (0.35–18.10)
Age, years		1.05 (1.03–1.07)	1.05 (1.03–1.07)		1.14 (1.11–1.17)	1.13 (1.10–1.16)
Ex-smoker		1.08 (0.95–1.22)	1.09 (0.96–1.23)		0.94 (0.35–2.51)	0.97 (0.36–2.58)
Current smoker		1.27 (1.17–1.39)	1.25 (1.14–1.37)		0.76 (0.11–5.39)	0.70 (0.10–4.99)
Alcohol (yes)		0.95 (0.87–1.03)	0.92 (0.85–1.00)		1.04 (0.91–1.22)	1.04 (0.90–1.22)
Exercise (yes)		0.89 (0.82–0.97)	0.91 (0.83–0.99)		1.08 (0.86–1.35)	1.08 (0.86–1.35)
SBP × 10 mm Hg		1.16 (1.13–1.20)	1.09 (1.06–1.12)		1.13 (1.06–1.20)	1.06 (1.00–1.13)
FBS × 10 mg/dL		0.99 (0.97–1.02)	0.98 (0.96–1.01)		1.00 (0.95–1.06)	0.99 (0.94–1.05)
TC × 10 mg/dL		1.06 (1.05–1.07)	1.04 (1.02–1.05)		1.00 (0.98–1.02)	0.99 (0.97–1.01)
**Mediators (2002**–**2004)**						
SBP × 10 mm Hg			**1.19 (1.16–1.22)**			**1.23 (1.17–1.30)**
FBS × 10 mg/dL			1.03 (1.02–1.04)			1.04 (0.99–1.08)
TC × 10 mg/dL			1.03 (1.02–1.04)			1.02 (1.00–1.05)

ASCVD, atherosclerotic cardiovascular disease; BMI, body mass index; CI, confidence interval; FBS, fasting blood sugar; HR, hazard ratio; SBP, systolic blood pressure; TC, total cholesterol.

A classical approach was used to estimate the direct and indirect effects of BMI on ASCVD. In men, the total effect of BMI on ASCVD incidence was 0.06 per BMI unit increase. Direct BMI effect on ASCVD was 0.04 (HR 1.04) when the continuous metabolic mediators SBP, FBS and TC were included in the model. In the classic approach when mediators were continuous variables, indirect effect was 0.02 (HR 1.02) using difference method. If this value is exchanged to proportion of mediated, 27.1% was mediated by mediators. In detail, the proportion mediated was largest in total stroke with 60.4%, while IHD was mediated by 21.5% (Table 3). Also in women, direct effect and indirect effect was estimated using the difference method. The proportion of indirect effect mediated by mediators was 33.3% in total ASCVD and was highest in AMI, which was 47.2% (Table 3).

**Table 3.** Direct and indirect effects of BMI on ASCVD using difference method in men and women: exposure in continuous and mediators in continuous

	ASCVD	Stroke			IHD		

		All	Ischemic	Hemorrhagic	All	AMI	Angina

	Parameter	Parameter	Parameter	Parameter	Parameter	Parameter	Parameter
Men							
Total effect (TE)	0.06	0.04	0.06	0.01	0.06	0.057	0.06
Direct effect (DE)	0.04	0.02	0.03	−0.02	0.06	0.04	0.05
Indirect effect (TE-DE)	0.02	0.02	0.03	0.03	0.01	0.01	0.01
% mediated	27.1	60.4	43.8	NE	13.5	21.5	12.6

Women							
Total effect (TE)	0.07	0.04	0.09	−0.00	0.09	0.03	0.01
Direct effect (DE)	0.05	0.03	0.06	−0.03	0.07	0.02	0.08
Indirect effect (TE-DE)	0.02	0.02	0.03	0.02	0.02	0.03	−0.07
% mediated	33.3	35.4	30.5	NE	20.4	47.2	NE

AMI, acute myocardial infarction; ASCVD, atherosclerotic cardiovascular disease; BMI, body mass index; FBS, fasting blood sugar; IHD, ischemic heart disease; NE, not estimated due to small sample or insignificant results; SBP, systolic blood pressure; TC, total cholesterol.

Total effect: adjusted for age, smoking, alcohol, exercise, systolic blood pressure (SBP), fasting glucose, and total cholesterol at baseline (’92 and ’94); Direct effect: adjusted for age, smoking, alcohol, exercise, SBP, fasting glucose, and total cholesterol at baseline plus mediators (SBP, FBS, and TC) (’02 and ’04); % mediated: (TE-DE)/TE * 100.

Table 4 describes the results of mediation analysis using the classic approach when exposure and mediator are both binary variables. The total effect of obesity on ASCVD was 0.23. The total effect was decomposed to direct and indirect effects. In men, the direct effect of obesity on ASCVD was 0.16 (HR 1.17) and the indirect effect was 0.07 (HR 1.07). The proportion of indirect effect was 30.7% among the total effect in ASCVD. The proportion of indirect effect was largest in total stroke, which was 48.8% (Table 4). In women, the total effect of obesity on ASCVD was 0.36 (HR 1.43), and the direct effect with mediator adjustment was 0.20 (HR 1.22). The total effect minus the direct effect was 0.16 (HR 1.17). Also in women, the proportion mediated by the indirect effect was largest for stroke (66.5%).

**Table 4.** Direct and indirect effects of obesity on ASCVD using difference method in men and women: exposure in binary and mediators in binary

	ASCVD	Stroke			IHD		

		All	Ischemic	Hemorrhagic	All	AMI	Angina

	Parameter (B)	Parameter (B)	Parameter (B)	Parameter (B)	Parameter (B)	Parameter (B)	Parameter (B)
Men							
Total effect (TE)	0.23	0.20	0.28	0.12	0.22	0.17	0.20
Direct effect (DE)	0.16	0.10	0.17	−0.02	0.18	0.11	0.17
Indirect effect (TE-DE)	0.07	0.10	0.11	0.14	0.04	0.05	0.04
% mediated	30.7	48.8	39.5	NE	18.5	32.6	17.5

Women							
Total effect (TE)	0.36	0.16	0.21	0.10	0.53	0.68	0.62
Direct effect (DE)	0.20	0.05	0.08	−0.03	0.39	0.48	0.49
Indirect effect (TE-DE)	0.16	0.11	0.14	0.12	0.14	0.21	0.13
% mediated	43.9	66.5	63.1	NE	26.2	30.0	21.0

AMI, acute myocardial infarction; ASCVD, atherosclerotic cardiovascular disease; IHD, ischemic heart disease; NE, not estimated due to small sample or insignificant results.

Total effect: adjusted for age, smoking, alcohol, exercise, systolic blood pressure (SBP), fasting glucose, and total cholesterol at baseline (’92 and ’94); Direct effect: adjusted for age, smoking, alcohol, exercise, SBP, fasting glucose, and total cholesterol at baseline plus mediators (hypertension, diabetes, and dyslipidemia) (’02 and ’04); % mediated: (TE-DE)/TE * 100.

The counterfactual method was used to estimate the direct and indirect effects of BMI on ASCVD. Table 5 and Table 6 describe the relationships between baseline characteristics and mediators and their associations with total and subtypes of ASCVD development in men and women. In both men and women, the direct effect of high BMI on the development of ASCVD was greater than the indirect effect. The percentage of excess HR of BMI mediated by hypertension, diabetes, and dyslipidemia were 15.2%, 17.0%, and 1.7%, respectively, on ASCVD in men (Table 5). Similar results were shown in women: the percentage of excess HR of BMI mediated by hypertension, diabetes, and dyslipidemia were 12.4%, 9.5%, and 7.9%, respectively (Table 6).

**Table 5.** Mediators of the obesity on stroke and IHD risk among men: decomposing the direct and indirect effects

Decomposing effect of mediators	ASCVD	Total stroke	Ischemic	Hemorrhagic	IHD
		
	HR (95% CI)	HR (95% CI)	HR (95% CI)	HR (95% CI)	HR (95% CI)
Hypertension					
Crude effect	1.36 (1.31–1.42)	1.31 (1.22–1.41)	1.45 (1.31–1.61)	1.21 (1.02–1.44)	1.35 (1.28–1.43)
Natural direct effect	1.33 (1.28–1.38)	1.27 (1.20–1.35)	1.41 (1.29–1.53)	1.12 (0.98–1.27)	1.32 (1.26–1.39)
Natural indirect effect	1.04 (1.04–1.05)	1.06 (1.05–1.08)	1.06 (1.04–1.08)	1.09 (1.06–1.13)	1.02 (1.01–1.03)
Total effect	1.39 (1.34–1.44)	1.35 (1.27–1.43)	1.49 (1.38–1.62)	1.22 (1.07–1.39)	1.35 (1.29–1.41)
% of mediated (SE)	15.2	22.0	18.0	47.6	6.9
Diabetes					
Crude effect	1.33 (1.28–1.37)	1.28 (1.20–1.35)	1.38 (1.27–1.50)	1.16 (1.03–1.32)	1.29 (1.23–1.35)
Natural direct effect	1.30 (1.26–1.34)	1.27 (1.20–1.34)	1.38 (1.28–1.49)	1.15 (1.02–1.29)	1.27 (1.22–1.32)
Natural indirect effect	1.05 (1.04–1.05)	1.05 (1.03–1.06)	1.06 (1.04–1.07)	1.04 (1.02–1.06)	1.05 (1.04–1.05)
Total effect	1.36 (1.32–1.40)	1.32 (1.26–1.39)	1.45 (1.35–1.56)	1.19 (1.06–1.33)	1.33 (1.28–1.38)
% of mediated (SE)	17.0	17.6	16.7	22.2	18.0
Dyslipidemia					
Crude effect	1.41 (1.34–1.48)	1.31 (1.20–1.42)	1.46 (1.30–1.64)	1.19 (1.01–1.41)	1.39 (1.30–1.49)
Natural direct effect	1.39 (1.33–1.44)	1.32 (1.24–1.41)	1.46 (1.33–1.60)	1.25 (1.09–1.43)	1.37 (1.30–1.44)
Natural indirect effect	1.00 (1.00–1.01)	1.00 (1.00–1.01)	1.01 (1.00–1.01)	1.00 (0.99–1.01)	1.01 (1.00–1.01)
Total effect	1.39 (1.34–1.45)	1.33 (1.24–1.42)	1.47 (1.34–1.61)	1.25 (1.09–1.43)	1.38 (1.31–1.45)
% of mediated (SE)	1.7	1.3	1.7	0.3	2.6

ASCVD, atherosclerotic cardiovascular disease; CI, confidence interval HR, hazard ratio, adjusted for age, smoking, alcohol, and exercise; IHD, ischemic heart disease; SE, standard error.

**Table 6.** Mediators of the obesity on stroke and IHD risk among women: decomposing the direct and indirect effects

Decomposing effect of mediators	ASCVD	Total stroke	Ischemic	Hemorrhagic	IHD
		
	HR (95% CI)	HR (95% CI)	HR (95% CI)	HR (95% CI)	HR (95% CI)
Hypertension					
Crude effect	1.63 (1.39–1.91)	1.31 (1.02–1.68)	1.12 (0.66–1.92)	1.12 (0.61–2.05)	2.03 (1.63–2.54)
Natural direct effect	1.61 (1.39–1.86)	1.30 (1.03–1.64)	1.20 (0.76–1.90)	1.23 (0.76–2.00)	1.95 (1.58–2.41)
Natural indirect effect	1.05 (1.02–1.09)	1.06 (1.00–1.12)	1.15 (1.00–1.33)	1.25 (1.06–1.48)	1.00 (0.96–1.04)
Total effect	1.69 (1.47–1.94)	1.38 (1.11–1.71)	1.38 (0.91–2.10)	1.54 (1.00–2.37)	1.96 (1.60–2.40)
% of mediated (SE)	12.4	19.9	4.83	57.4	0.5
Diabetes					
Crude effect	1.51 (1.30–1.76)	1.20 (0.94–1.52)	1.21 (0.75–1.94)	1.50 (0.96–2.34)	1.73 (1.38–2.17)
Natural direct effect	1.49 (1.29–1.72)	1.19 (0.95–1.50)	1.21 (0.77–1.90)	1.46 (0.94–2.26)	1.70 (1.37–2.12)
Natural indirect effect	1.03 (1.01–1.06)	1.03 (1.00–1.07)	1.10 (1.00–1.20)	0.99 (0.95–1.04)	1.04 (1.00–1.07)
Total effect	1.54 (1.34–1.77)	1.23 (0.99–1.54)	1.33 (0.88–2.00)	1.45 (0.95–2.22)	1.77 (1.44–2.17)
% of mediated (SE)	9.5	17.6	35.2	−2.0	8.4
Dyslipidemia					
Crude effect	1.40 (1.13–1.75)	1.03 (0.73–1.46)	0.98 (0.48–1.99)	1.11 (0.57–2.18)	1.85 (1.35–2.55)
Natural direct effect	1.51 (1.27–1.79)	1.19 (0.91–1.54)	1.27 (0.78–2.07)	1.17 (0.68–2.00)	1.83 (1.42–2.37)
Natural indirect effect	1.03 (1.00–1.06)	1.05 (1.00–1.09)	1.08 (0.99–1.18)	1.02 (0.94–1.10)	1.01 (0.97–1.05)
Total effect	1.55 (1.32–1.83)	1.24 (0.97–1.60)	1.37 (0.86–2.18)	1.19 (0.71–2.00)	1.85 (1.44–2.37)
% of mediated (SE)	7.9	22.5	27.1	10.8	2.5

ASCVD, atherosclerotic cardiovascular disease; CI, confidence interval HR, hazard ratio, adjusted for age, smoking, alcohol, and exercise; IHD, ischemic heart disease; SE, standard error.

## DISCUSSON

We examined the association between baseline BMI and the risk of ASCVD among Korean young adults who were aged 20 to 39 years in 1992 or 1994 along with the metabolic mediators measured in 2002 and 2004. In both men and women, higher BMI had strong, progressive associations with ASCVD.

We also decomposed the effects of BMI on ASCVD into total, direct, and indirect. We conceptualized the total effects as the effects from covariate-adjusted baseline BMI to ASCVD, direct effects as effects from baseline BMI to ASCVD after additionally adjusted for intermediate variables, and indirect effects as the coefficient of the exposure in the mediator model times the coefficient of the mediator in the outcome model.

Our study conducted two approaches to the effects of obesity on ASCVD: the classic method (Table 3 and Table 4) and the counterfactual method (Table 5 and Table 6). The direct effect of BMI on ASCVD was greater than the indirect effect.

Until now, it was only understood that high baseline SBP or hypertension was simply associated, without a specific mechanism, since previous studies reported that SBP was the main risk factor of hemorrhagic stroke.^21^^,^^22^ Our results support the result from previous studies that obesity was very effective in raising the risk of hemorrhagic stroke through increasing SBP as an intermediate mediator.

Using a counterfactual approach, the total effect was decomposed to natural direct effect and natural indirect effect, and based on these effects, the proportion mediated was estimated. On hemorrhagic stroke, the proportion mediated by hypertension of men and women was 47.6% and 57.4% respectively. Results from our study could be evidence to predict the intervention effect of managing obesity and hypertension. Further studies comparing cost per effect of managing obesity and SBP using economic evaluation method are required to quantitatively measure the effect of intervention and to make better prediction strategies. In our study, the percentage of excess HR was lower than previous findings. Lu and colleagues reported that metabolic mediators explain about half of the adverse effects of high BMI on coronary heart disease.^23^ The differences between our study and the previous study can be explained in several ways. First, they used baseline measurements of mediators, while our study used mediators measured after 10 years from baseline. Also, racial differences in the mechanisms of obesity and heart disease between the studies might account for the inconsistent results. Further research is needed to enhance understanding of the association between obesity and heart disease in different populations. Our study found that managing obesity in young adults is expected to help prevent heart disease in their middle age. Also, obese young adults still have a chance to reduce their heart disease risk in their middle age, if they manage their metabolic mediators well in their later life.

Because early management and intervention plays an important role in preventing CVD, increasing number of researchers have focused on childhood as an early period of vulnerability to insults, with the assumption that childhood vulnerability may elevate risks of CVD in adulthood.^24^^–^^26^ However, it has not been studied whether this theory applies to young adults. In other words, it remains to be investigated whether young adulthood can be a critical period for disease risk in adulthood, including the elderly years. The life course approach to health emphasizes temporal, social perspectives on individuals’ or cohort’s life experiences across generations for clues to current patterns of health outcomes. Not only the period in utero and early infancy, but also childhood and adolescence, are conceptualized as critical periods of growth and development in the human life cycle, when early risk factors do damage to long-term health. Early risk exposures in critical periods interact with later modifiers to have synergetic effects on health outcomes in middle age.^27^^–^^29^ With our study design, we aimed to apply the life course approach to ASCVD management, assuming that young adults aged 20–29 may be another critical period in the life course. We conceptualized baseline BMI as an early risk factor and metabolic mediators measured after 10 years as later-life factors. The results of this study suggest that young adulthood can be also a critical period for cardiovascular health in later adulthood.

The American Heart Association (AHA) defined “ideal cardiovascular health” as the simultaneous presence of three physiologic factors: total cholesterol, blood pressure, and fasting glucose without medication, along with four health behaviors: nonsmoking, normal BMI, adequate physical activity, and healthy diet.^30^ We incorporated the AHA-defined variables and compared the values between the two study time points: one at the study baseline (young adulthood) and the other in the midst of the study follow-up (later adulthood). The level of TC showed the largest increase between the baseline and intermediate periods of follow-up. Moreover, compared to women, higher levels of SBP or TC had greater risks for ASCVD in men (Table 2). Although the effects of changing dietary habits towards a more westernized style might explain the dramatic changes in cholesterol levels (Table 1),^31^^,^^32^ more specific studies are needed to clarify the explanations. Nevertheless, another finding that baseline FSG and TC in women were not related to ASCVD may be attributed to the fact that most of the women in this study were premenopausal, indicating that they were at relatively lower risk of CVD.^33^

### Strengths

The main strength of this study is the long-term follow-up period of a large cohort with over 200,000 young adults, allowing us to capture changes in disease risk factors over time. Moreover, the complete follow-up for ASCVD events allowed us to examine disease risk in detail across a wide range of BMI. Also, measurement error was minimized by using measured BMI and by using the averages from 1992 and 1994. It was also possible to evaluate the effects of the metabolic mediators by reexamining the same measurements in 2002 and 2004, after 10 years from the baseline.

### Limitations

Several limitations of our study should be acknowledged in terms of the follow-up period and representativeness of our participants. First, 23-year follow-up period is still a short term for assessing the effect on health outcomes in the elderly, considering that the Korean population is aging rapidly. Second, since only special subjects—civil servants and private school staff—were included in the study, we should be cautious about generalizing these findings. Moreover, we were not able to consider the interaction effect between our exposures and mediators, which will need to be discussed in further studies.

### Conclusion and recommendation

In conclusion, high BMI in young adults was an independent risk factor for CVD in their middle age. High BMI increased the risk of metabolic mediators in middle age, which explain about 20–30% of the adverse effects of high BMI on ASCVD. Our results suggest that ASCVD intervention should focus not only on primary prevention of obesity but also on hypertension, diabetes, and cholesterol.

## SUPPLEMENTARY MATERIAL

eTable 1. Pearson correlation between body mass index at baseline (1992–1994) and mediators (2002–2004)

eTable 2. The effect of body mass index at baseline (1992–1994) on mediators (2002–2004) after adjusted for age at baseline
