# Supplementary material for: Mediators of the Effect of Obesity on Stroke and Heart Disease Risk: Decomposing Direct and Indirect Effects
Source: J Epidemiol. 2023 Oct 5;33(10):514–20. doi: 10.2188/jea.JE20210476 (PMC10483103; doi:10.2188/jea.JE20210476)
Supplement: Supplementary file 1 [file je-33-514-s001.pdf]

eTable 1. Pearson correlation between body mass index at baseline (1992–1994) and mediators (2002–2004)

	Mediators					
	SBP		FBS		TC	
	r	*P* value	r	*P* value	r	*P* value
Men	0.2621	<0.0001	0.1998	<0.0001	0.1468	<0.0001
Women	0.2306	<0.0001	0.1447	<0.0001	0.1476	<0.0001

FBS, fasting blood sugar; SBP, systolic blood pressure; TC, total cholesterol.
Adjusted for age, smoking status, alcohol drinking, and exercise.

eTable 2. The effect of body mass index at baseline (1992–1994) on mediators (2002–2004) after adjusted for age at baseline

	Mediators					
	SBP		FBS		TC	
	B (se)	*P* value	B (se)	*P* value	B (se)	*P* value
Men	1.3935	<0.0001	1.7773	<0.0001	1.8748	<0.0001
Women	1.1608	<0.0001	0.7982	<0.0001	1.4660	<0.0001

FBS, fasting blood sugar; SBP, systolic blood pressure; TC, total cholesterol.
Adjusted for age, smoking status, alcohol drinking, and exercise.
